# Inner Fire: Building Competence and Resilience to Enable the Effective Management of Integrated Care Systems

**DOI:** 10.5334/ijic.5499

**Published:** 2020-04-01

**Authors:** Nicholas Goodwin

**Affiliations:** 1 Central Coast Research Institute, NSW, AU

**Keywords:** Integrated care, management, networks, leadership, competences, culture, partnerships

One of the core challenges for the successful adoption of integrated care systems around the world lies in the effectiveness of its management and leadership strategies – and of the skills and competencies of those managers and leaders tasked with implementing them. An effectively crafted integrated care programme or system is required for successful partnership working with people and communities, across professional groups, between organisations and sectors, and to multiple care settings. Such crafting requires significant management skill to facilitate partnerships to grow, endure and become legitimate.

In this edition of IJIC, an exploratory study by Miller and Stein report on the findings of a qualitative study based upon interviews with senior professionals, researchers, managers and policy-makers with extensive experience of implementing integrated care [1]. Given the lack of clarity on what ‘good’ management looks like, the authors sought to determine whether management skills and competences for integrated care should be perceived as different to that of ‘traditional’ care and, if so, in what areas such competences are needed and how to acquire them.

The results of Miller and Stein’s work indicate that if networks across professions and organisations are to be supported effectively then managers need to have many qualities – for example, an in-depth knowledge of the wider system in which they operate; being comfortable in distributing leadership and authority; understanding and embracing cultural differences; not being afraid to challenge the status quo; and operating with integrity and humility to foster trusted relationships. Feedback from research participants also indicated a lack of opportunities for managers to develop these core skills despite seeing them as fundamental to the process. Moreover, the findings indicate that managers supporting network-based activities are not ‘simple’ administrators but require significant leadership and negotiation skills.

One of the most useful discourses in Miller and Stein’s work is the differentiation between the strategic and operational aspects of integrated care management. There is often a dissonance between the two levels, specifically in strategic decisions that are made in ways that are not cognisant of operational reality, so leading to unrealistic expectations of the operational managers to deliver results. For example, in the lack of recognition to the time and effort required to build effective partnerships so they can reach the necessary level of maturity to demonstrate impact. Managers responsible for achieving strategic change through integrated care need to plan over an appropriate timescale and to base their actions on a coherent change management strategy [2,3].

The network manager – the ‘boundary spanner’ tasked with managing inter-professional and inter-organisational relations to ensure better care coordination – most often does not have the authority to make things happen and may quickly find themselves isolated. As Miller and Stein aptly describe, those that thrive often require an ‘inner fire’ – an in-built resilience to ‘weather the storms and persevere in their endeavours’. Investing in the skills and resilience of the network manager, therefore, is highly significant since the effectiveness of their role is not dependent on status, but on their skills as a committed, reliable and trusted facilitator [4]. However, as Miller and Stein point out, there remains inadequate investment – and a lack of understanding – in how to better prepare and support the skills of such managers.

The effective implementation of integrated care has many complexities, but Miller and Stein are undoubtedly correct that the lack of understanding and investment in management skills and competencies at this level represents an Achilles heel to the movement internationally. It may be observed that management training and support for integrated care is prevalent in many countries – for example, via charitable or commercial agencies providing consultant-based management training and support (e.g. in the UK and Canada) through to significant in-house training programmes in large managed care organisations (e.g. in the USA) to companies offering to fully take over integrated care management practices (e.g. in USA, UK, Spain and Germany). However, most of these focus on strategic management and leadership rather than building the necessary skills of the network manager working at a local level.

In reflecting on Miller and Stein’s conclusions it seems that there remains a common problem to many integrated care programmes in that they focus on organisational solutions as opposed to how care services can be better integrated and coordinated across a network of providers and professionals at the local level. Relative investment in the management skills required to make integrated care systems work is significantly unbalanced. To meet future needs, investment needs to be recalibrated so it comes much closer to the ‘front-line’. I suspect that the ‘inner fire’ of any operational manager working at a local level comes from their insatiable appetite to improve care outcomes for people. Yet, they often have to work in a hostile environment embedded with cultural and structural fragmentations that need a special type of resilience and specialist management skills. If integrated care strategies are to succeed we need to “feed the fire” and augment such skills or risk extinguishing the very flames necessary within managers who must provide the energy and commitment that is required.
